# Effect of vertical implant position on marginal bone loss: a randomized clinical trial

**DOI:** 10.1186/s12903-024-04480-7

**Published:** 2024-06-24

**Authors:** Morad Hedayatipanah, Hadi Kokabi Arasteh, Abbas Shokri, Behnaz Alafchi, Leila Shahsavand Baghdadi

**Affiliations:** 1 Department of Periodontics, Faculty of Dentistry, Hamadan University of Medical Sciences, Hamadan, Iran; 2 Department of Oral and Maxillofacial Radiology, School of Dentistry, Hamadan University of Medical Sciences, Hamadan, Iran; 3 Department of Biostatistics, School of Public Health, Hamadan University of Medical Sciences, Hamadan, Iran

**Keywords:** Marginal bone loss, Vertical implant position, Soft tissue thickness

## Abstract

**Objectives:**

One of the most important factors that has influence on dental implants success rate is marginal bone loss. The purpose of this study is to investigate the effect of the implant’s vertical position and the soft tissue’s thickness on the rate of marginal bone loss of the dental implant.

**Materials and methods:**

In this single-blind randomized clinical trial study, 56 implants placed in the posterior region of mandible of 33 patients (19 women, 14 men) were divided into two groups. The group of crestal (28 implants) and subcrestal (28 implants) implants, each group was divided into two sub-groups with soft tissue thickness of 2 mm and less than 2 mm (14 implants) and more than 2 mm (14 implants). The amount of marginal bone loss was measured by Scanora 5.2 program with radiographs Digital parallelism based on the effect of the vertical position of the implant, soft tissue thickness, three months after placement, and three months after loading implants (six months after implant placement).

**Results:**

The results showed that marginal bone loss in subcrestal implants is significantly more than crestal implants (p-value = 0.001), and also marginal bone loss in the soft tissue thickness group of 2 mm and less is significantly more than the group of soft tissue thickness more than 2 mm (p-value < 0.001). The amount of marginal bone loss three months after implant loading was significantly higher than three months after implant placement (p-value < 0.001).

**Conclusion:**

The implant’s vertical position and the soft tissue’s thickness around the implant are effective factors in the amount of marginal bone loss. Marginal bone loss is more in subcrestal implants and in cases with less soft tissue thickness. The time factor significantly affects the amount of marginal bone loss.

**Trial registration:**

this clinical trial was registered at Iranian Registry of Clinical Trials, registration number IRCT20120215009014N415, registration date 20,220,110, (https//en.irct.ir/trial/60,991)

## Background

Reconstruction of the edentulous space is one of the main fields of restorative dentistry. Throughout history, various techniques and materials have been used to reconstruct the edentulous space. In 1950, Bernmark introduced osseointegration into reconstructive dentistry. His studies showed that pure titanium could be attached to the bone in immobile contact with alveolar bone. This study resulted in a revolution in dentistry and the reconstruction of edentulous spaces. The success rate of dental implants is high, but it’s not 100%. Bone loss around the implant was one of the factors that caused the failure of dental implants. Many studies have been done on the factors affecting the success of dental implants, but the effects of some factors are still not completely clear [1–5].

In recent years, the replacement of lost teeth by implants has been done in full and partial form [6]. In studies, the success rate of dental implants in non-smokers has been reported to be 95.2–98.8% [7]. Symptoms such as peri-implantitis, mobility, crestal bone loss, radiolucency around the implant, persistent pain, infection, neuropathies, paresthesia, and interference with the mandibular canal cause the failure of dental implants [8]. The stability and absence of bone loss around the implant is an important factor in the survival rate of dental implants [9]. Factors such as surgical trauma, soft tissue thickness, biological width, types of connections between fixture and abutment, implant design in the crestal area, one-stage or two-stage implant placement, micro movement of abutment, placement and removing of abutment and microgap between implant connections- Abutments are effective in remodeling the bone around the implant [10]. Factors that affect marginal bone loss around the implant can be causes such as prosthetic factors [11], systemic factors (uncontrolled diabetes, smoking, etc.), local factors (periodontal diseases, poor hygiene), and implant characteristics (surface, length, diameter, and morphology) [10, 12–14]. Early detection of bone loss is essential because clinicians can perform preventive and corrective treatments faster [15]. During the first year after implant placement, marginal bone remodeling occurs [8, 16]. The amount of marginal bone loss around the functional implant during the first year for the success and survival of the implant is between 1 and 2 mm [8, 17, 18].

Early bone loss around dental implants is a significant concern that can negatively impact their long-term survival and prognosis. Impact of Early Bone Loss on Implant Survival:

Compromised Osseointegration: Osseointegration, the fusion of the implant with the jawbone, is crucial for implant stability. Studies have shown that excessive bone loss in the first few months (especially exceeding 1.5 mm) can hinder osseointegration, leading to implant loosening and potential failure later [19, 20]. Increased Risk of Peri-implantitis: Early bone loss creates pocket around the implant, allowing bacteria to accumulate and trigger peri-implantitis [21].

One of the influencing factors on the marginal bone loss around the implant is its vertical position (crestal or subcrestal (1–2 mm)), which has not reached common results in different articles about the effect of the vertical position of the implant on the marginal bone loss [22]. Therefore, this study aims to investigate the effect of the vertical position of the implant and soft tissue thickness on the marginal bone loss around it.

## Materials and methods

### Study design

This study is a randomized single-blind clinical trial composed of two groups (vertical dental implant crestal and subcrestal) of 28 dental implants each, and two subgroups (soft tissue thickness of 2 mm and less than 2 mm (≤ 2) and with a soft tissue thickness greater than 2 mm (> 2 )),14 dental implant each, and it was conducted following the Consolidated Standard of Reporting Trials(CONSORT) [23]. This clinical trial was registered at Iranian Registry of Clinical Trials, registration number: IRCT20120215009014N415, registration date: 2022-01-10, (https://en.irct.ir/trial/60991). This study was approved as an interventional clinical trial in the ethics committee of Hamadan University of medical sciences (IR.UMSHA.REC.1400.694) and guidelines of the Helsinki Declaration were followed.

### Patient population

After selecting the patients who met the inclusion and exclusion criteria, the steps and objectives of the study were fully explained to the patients, and informed consent was obtained from them. The study population of this research was the number of 56 implants for 33 patients referred to the implant department of Hamedan Dental School. The participants were randomly divided into crestal (at the level of the crest bone) and subcrestal (1–2 mm more apical than the crest bone) implant groups. After measuring the soft tissue thickness, each group was divided into two groups with a soft tissue thickness of 2 mm and less than 2 mm (≤ 2) and with a soft tissue thickness greater than 2 mm (> 2) (in each group 14 implants) that a total of 56 implants were included in the study, the buccolingual, mesiodistal and apicocoronally bone thickness of the implant placement area was measured by CBCT radiography.

All surgical procedures were performed by the same experienced periodontist. An experienced prosthetist performed the prosthetic procedures for all participants.

#### Inclusion criteria

patients between 20 and 70 years old, systemically healthy, candidates for dental implants, delayed implant placement (in the restored area, at least three months after tooth extraction), implant placement with Flap in the posterior region of the mandible.

#### Exclusion criteria

Smoking, use of drugs affecting bone remodeling (bisphosphonate), presence of systemic disease affecting bone loss, uncontrolled diabetes, keratinized gingiva less than 2 mm, performing GBR (guided bone regeneration), presence of uncontrolled periodontitis and poor oral hygiene before the surgery (grade 2 or 3, Loe and Silness gingival index and plaque index > 15% according to O’Leary index), Immediate implant loading and fresh socket implant.

### The dental implants used endosteal root form

We used bone level type Korean SNUCONE Implants (AF + B Type,11 ° Tapered Hex, sandblasting with large grit, and acid etching (SLA)) and fixture diameter between 4 and 4.3 mm and length between10-12 mm.

**Fig. 1:**
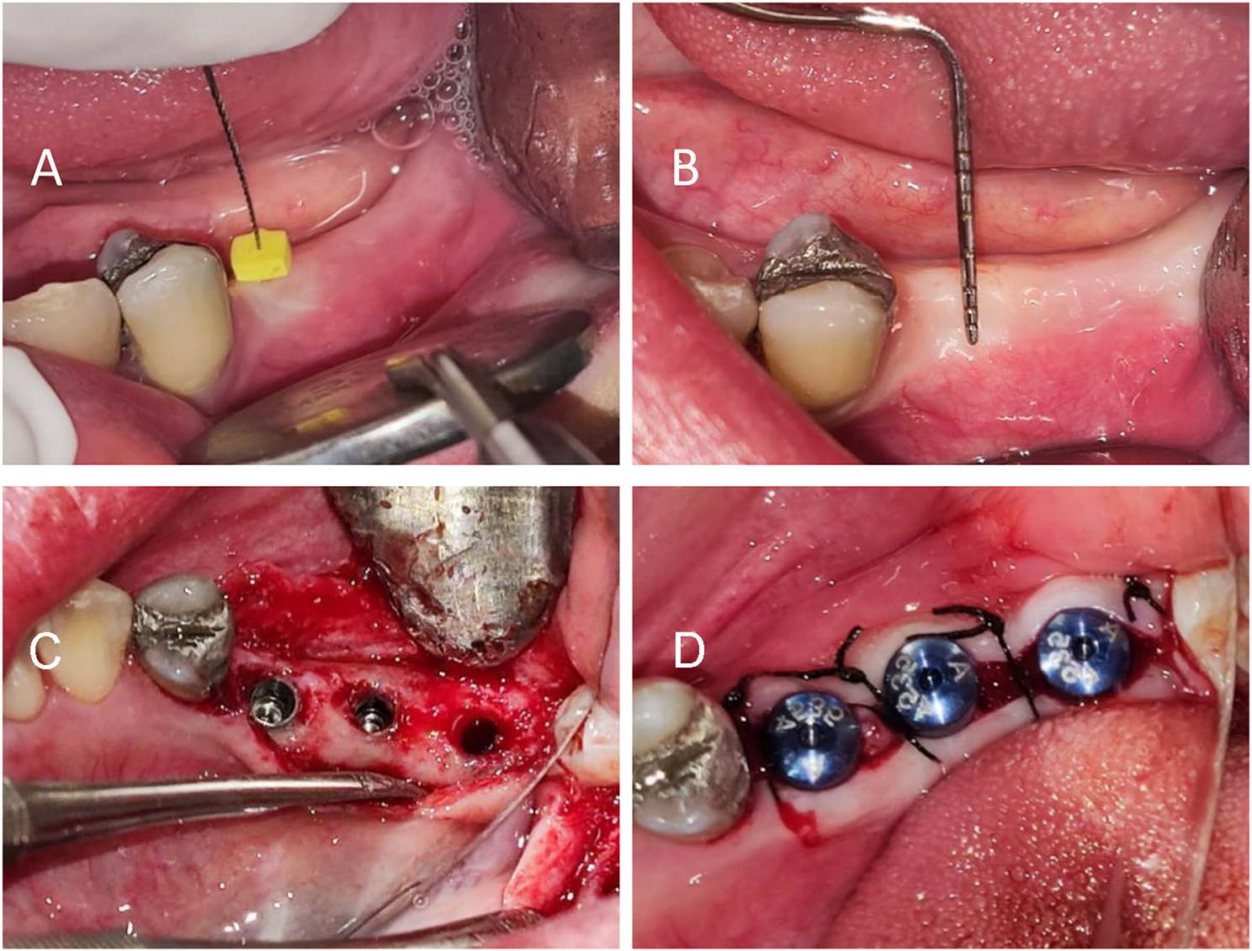
**(A)** Soft tissue thickness measurement **(B)** Keratinized gingiva measurement **(C)** Implant placement **(D)** Three months after implant placement and healing abutment closure

The patients were asked to use 0.12% chlorhexidine mouthwash (Chlorhexidine SHD 0.12%, Behsa, Tehran, Iran) for one minute before surgery. After injection of 2% lidocaine with 1/100,000 epinephrine (Persocaine-E, Darou Pakhsh Mfg. Co., Tehran, Iran) using infra-alveolar nerve block and infiltration for anesthesia, the thickness of patients’ soft tissue was measured by endodontic k-file No.15(Fig. 1-A) and endometrium. Also, the amount of keratinized gingiva was measured by a probe O the University of Michigan (Fig. 1-B). After the incision was performed with a No. 15 blade and the full-thickness flap retracted, A round surgical bur was used to create a flat bony surface; drilling was performed according to the protocol of the implant manufacturer, to prevent excessive heat during drilling, abundant washing with normal saline is used and inserted the implants with a torque of 30–35 N/cm, and the cover screw was placed (Fig. 1-C), and finally, the surgical site was sutured. All patients received 1 g of amoxicillin and 500 gm metronidazole (Amoxicillin 500 mg, metronidazole 250 mg; KosarDaru, Tehran) and use 0.12% chlorhexidine mouthwash for plaque control (Chlorhexidine SHD 0.12%, Behsa, Tehran, Iran) twice a day for one week.

**Fig. 2:**
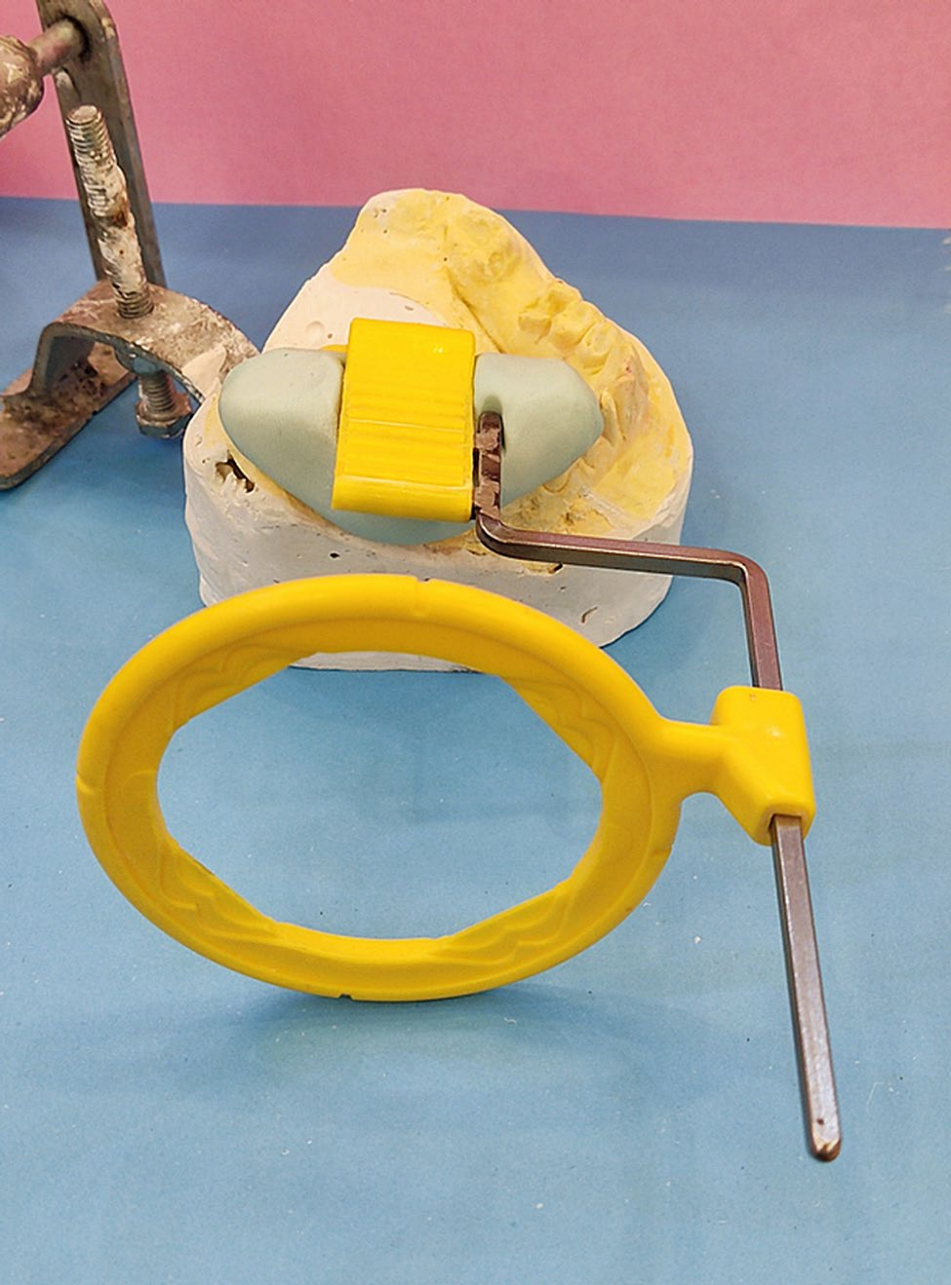
Film holder and putty index for the repeatable position for taking radiographs

**Fig. 3:**
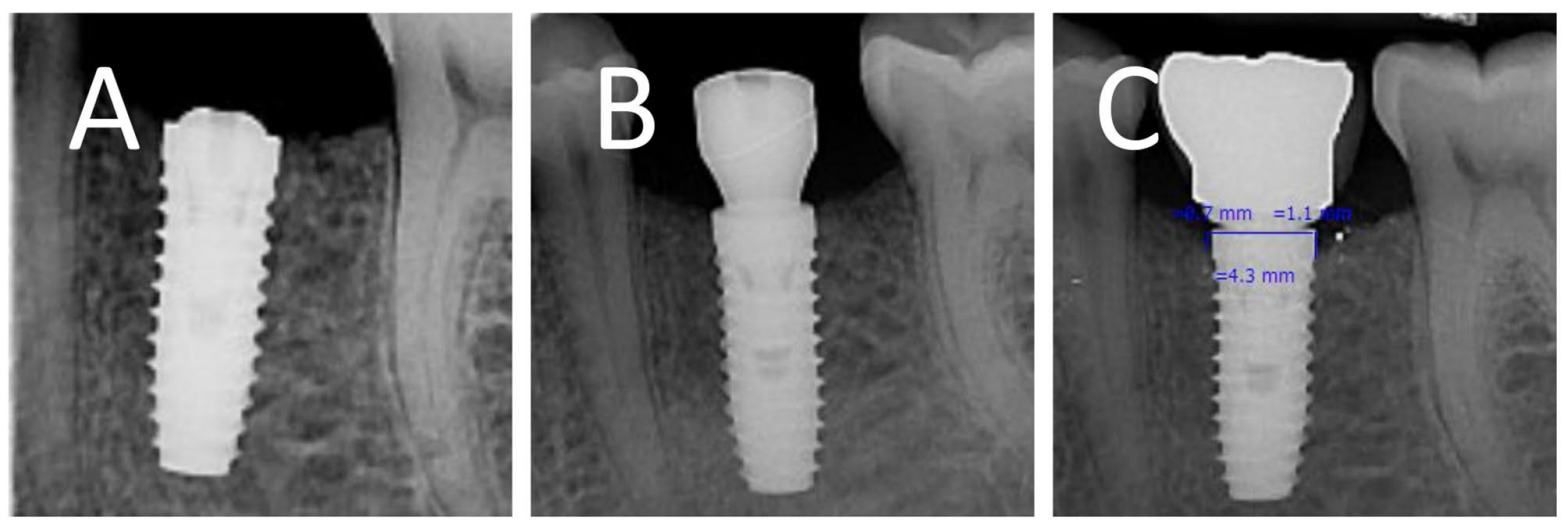
Crestal implant: **(A)** baseline **(B)** three months after placement **(C)** three months after loading

**Fig. 4:**
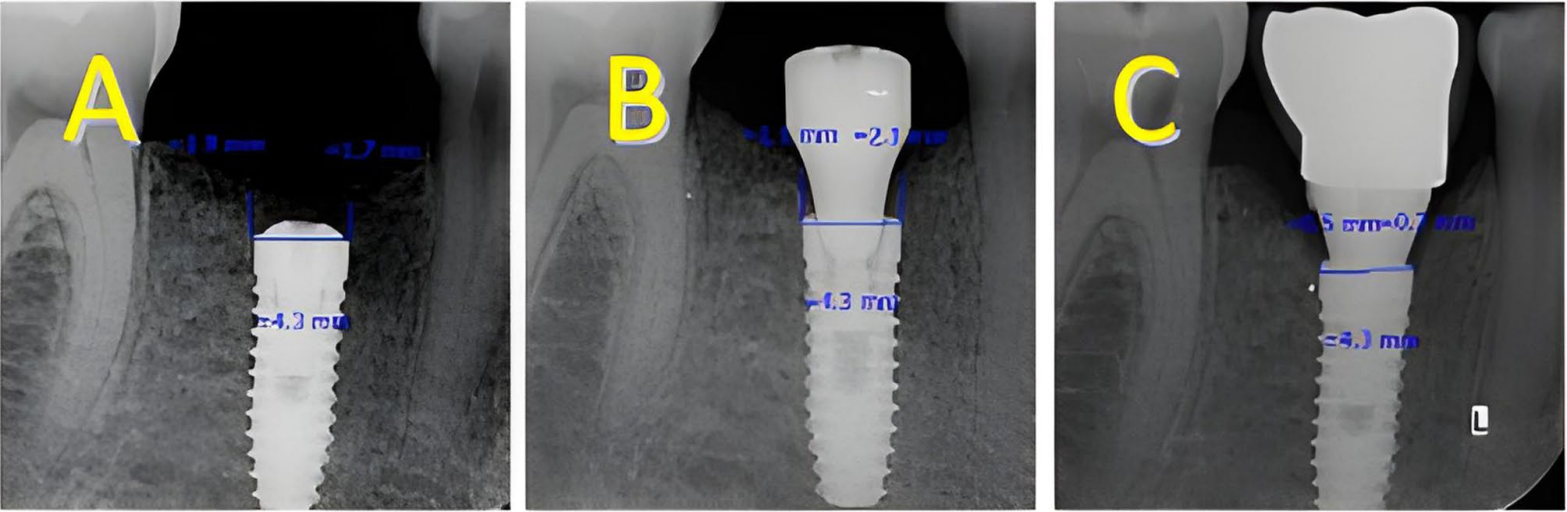
Subcrestal implant: **(A)** baseline **(B)** three months after placement **(C)** three months after loading

Suture materials were removed after 7 days. To take parallel periapical radiographs at the same angle, we made a stent with putty and created an index for a film holder (RINN) in the stent. The tube was adjusted according to the path that putty dictated to the film holder so that the position could be repeated (Fig. 2), then digital periapical radiography was prepared in a parallel way from the Implants. Radiographs were prepared by Minray device (Soredex, Tusuula, Findland) and by size 2 PSP film, Scan exam one (KAVO, Tusuula, Findland) with conditions of KVP = 60, MA = 6, T = 0.25 s. We used Scanora software (soredex, Tusuula, Findland) to measure marginal bone loss in the mesial and distal. Thus, in the parallel radiographic image, bone loss was measured from two points on the mesial and distal sides, from the module crest or the implant platform as a fixed index to the bone crest. In the baseline, marginal bone was coronally (1–2 mm) from the implant platform in the subcrustal method. It was at the level of implant platform in the crestal method. Amount of marginal bone loss in the mesial and distal of the implant in 3 months after placing the implant (healing abutment placing session) (Fig. 1-D) (two-stage surgical protocol) and 6 months after implant placement (3 months after implant loading) was the first contact area between the bone and implant in the mesial and distal area to implant platform defines the level of loss were checked through parallel periapical radiography (Figs. 3, 4).

The restorative protocol of all implants includes the conventional protocol (the prosthesis was placed 90 days after implant placement, including 14 days with healing cap) and we used prefabricated straight abutment, CHS: 7 mm (minimum vertical cantilever), Prosthetic screw torque: 35/screw access: central fossa, Restoration: Zirconium, Cemented: Zinc Phosphate.

The data were analyzed using appropriate statistical tests such as independent t-test, analysis of variance of repeated measures, dependent t-test, and chi-square test. It should be noted that the assumption of normality of the data was checked and used using the Shapiro-Wilks test. All calculations were done using SPSS version 24 software. The significance level of the test is considered to be 0.05 (*p* < 0.05) and a test power of 90%.

## Result

This randomized clinical trial randomly evaluated 33 patients (19 women, 14 men), 43% were men, and 57% were women. The average age of the patients was 43.00 ± 10.71. We use 56 implants in patients.

Information about implants and demographics is in Table 1.

**Table 1:** Demographic and Implants data

Implant size	Implant length	Surgical site	Average age	Male	Female
4–4/3 mm	10–12 mm	Posterior mandible	43.00 ± 10.71	43%	57%

The Shapiro-Wilks test showed that the marginal bone loss variable had a normal distribution.

The mean and standard deviation of the variable of marginal bone loss by group variables and soft tissue thickness are presented in Table 2. Repeated measure ANOVA was used to investigate the effect of soft tissue thickness on marginal bone loss in near and far distances. The equality test of box covariance matrices was insignificant (*P* = 0.660), which shows that this hypothesis has been verified. Also, the test of sphericity was not significant.

**Table 2:** Descriptive statistics of marginal bone resorption by group and tissue variables

			Group			
			Crest		Subcrest	
			Mean	SD	Mean	SD
Marginal Bone Loss at 3months	Tissue	<=2	− 0.40	0.26	− 0.77	0.54
		> 2	− 0.11	0.65	− 0.32	0.48
Marginal Bone Loss at 6months	Tissue	<=2	− 0.91	0.31	-1.58	0.55
		> 2	− 0.29	0.65	− 0.77	0.42

**Fig. 5:**
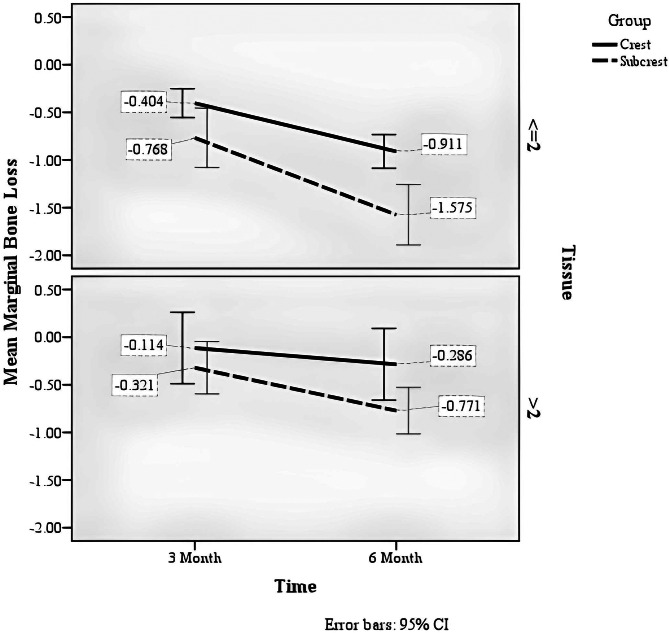
Average marginal bone loss in three- and six-month follow-up by group and tissue variable

**Fig. 6:**
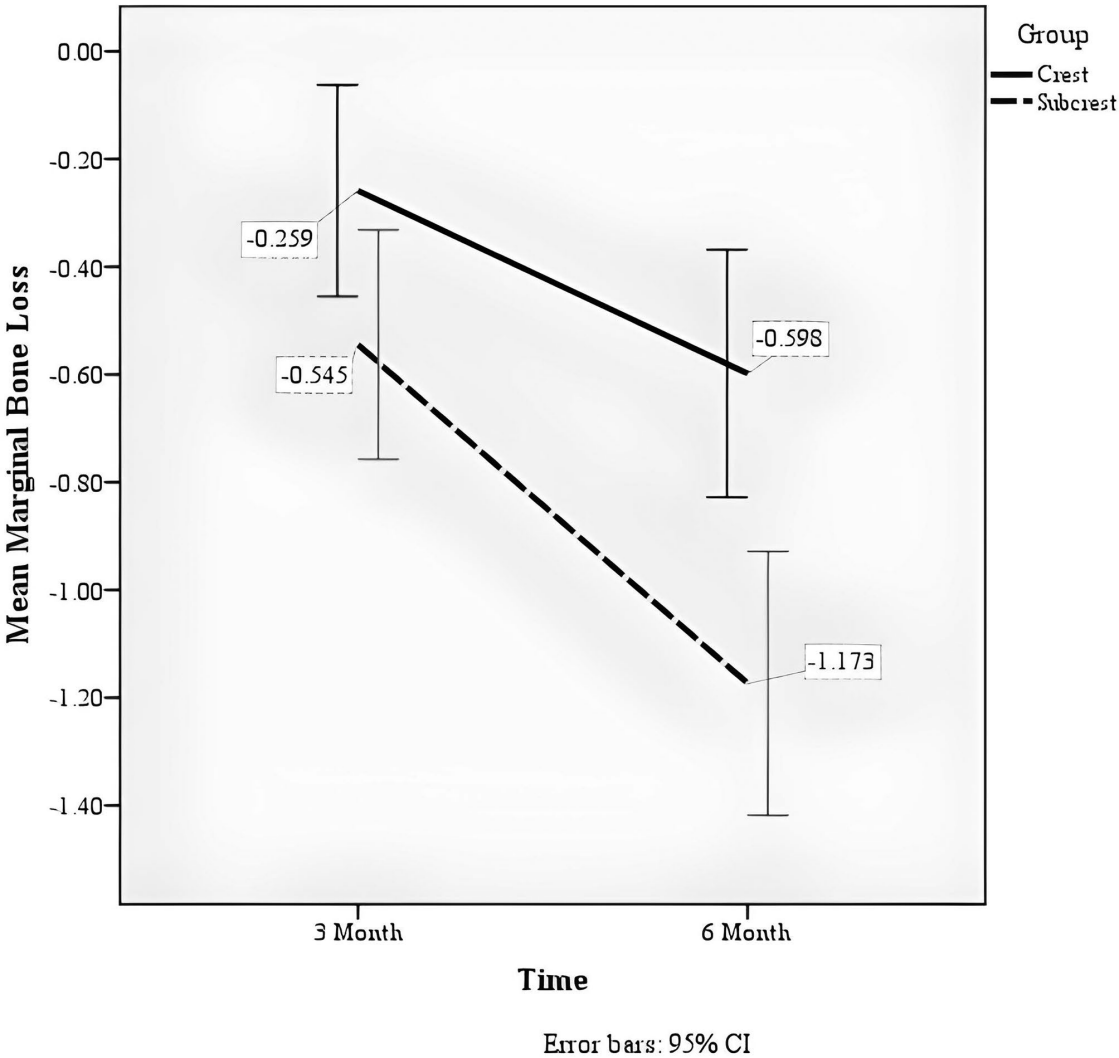
Average marginal bone loss in three- and six-month follow-up by group variable

**Fig. 7:**
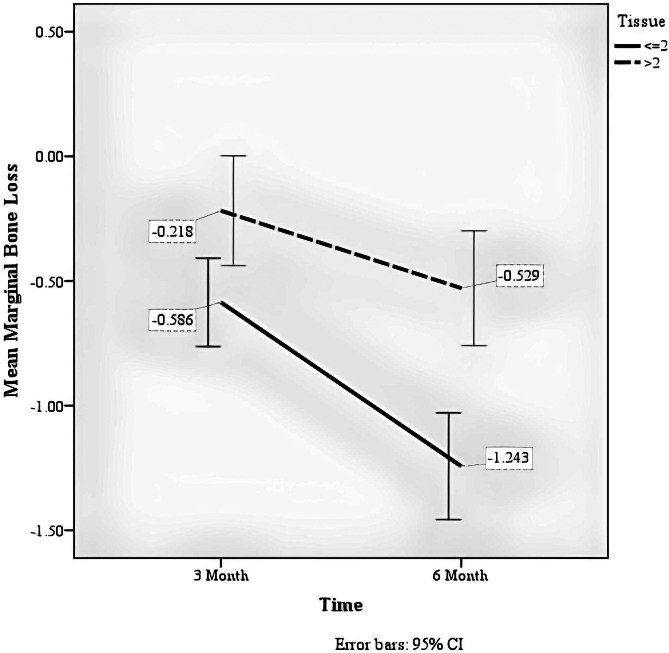
Average marginal bone loss in three- and six-month follow-up by tissue variable

The repeated measures ANOVA test showed the following results:

1. The group factor (crest or subcrest) significantly affected marginal bone loss (F = 11.867, *p* = 0.001), so the marginal bone in the subcrestal group had more loss than the crestal group.
2. The soft tissue thickness factor had a significant effect on marginal bone loss (F = 18.759, *p* < 0.001), so that the marginal bone in the soft tissue thickness group of 2 mm or less had more loss than the soft tissue thickness group of more than 2 mm.
3. The time factor significantly affected marginal bone loss (F = 111.770, *p* < 0.001) so that marginal bone loss was higher in six months than in three months (Fig. 5).
4. There was a significant interaction between the time factor and the group factor (F = 9.985, *p* = 0.003); the amount of marginal bone loss at different times depends on the study group. You can see the existence of this interaction in Fig. 6.
5. There was a significant interaction between the time and soft tissue thickness factors (F = 14.320, *p* < 0.001). The amount of marginal bone loss at different times depends on the soft tissue thickness (Table 3). You can see the existence of this interaction in Fig. 7.

**Table 3:** Comparison of marginal bone resorption between study groups

	Group				
	Crest		Subcrest		*P*-value^†^
	Mean	SD	Mean	SD	
Marginal Bone Loss at 3 months	− 0.26	0.51	− 0.54	0.55	0.048
Marginal Bone Loss at 6 months	− 0.60	0.59	-1.17	0.63	0.001

^†^Independent-Samples T-Test

Due to the interaction effect between follow-up time and group variables and soft tissue thickness, a comparison between the study groups and soft tissue thickness was made by separate follow-up times.

There was a statistically significant difference in marginal bone loss in three months and six months between the two study groups (*p* < 0.05). Still, the difference in this variable between the two groups was greater in the six-month follow-up than in the three-month follow-up (Table 4).

**Table 4:** Comparison of marginal bone resorption between two groups of soft tissue thickness

	Tissue				
	<=2		> 2		*P*-value^†^
	Mean	SD	Mean	SD	
Marginal Bone Loss at 3 months	− 0.59	0.46	− 0.22	0.57	0.010
Marginal Bone Loss at 6 months	-1.24	0.55	− 0.53	0.59	< 0 0.001

^†^Independent-Samples T-Test

There was a statistically significant difference in marginal bone loss in three months and six months between the two groups of soft tissue thickness (*p* < 0.05). Still, the difference between the two groups was greater in the six-month follow-up than in the three-month follow-up (Table 1).

## Discussion

Tooth loss affects the aesthetic, ability to chew and speak. Today, one of the main ways to restore toothless areas and improve function and aesthetics are intraosseous dental implants, used as augmentation [24, 25]. In various surveys, the success rate of dental implants has been reported to be excellent, and the results of these surveys have stated that this treatment is safe, predictable, and reliable [26]. However, some factors, such as peri-implantitis, infection, etc., cause implant failure [8, 27].

The stability and absence of bone loss around the implant are an important factor in the survival rate of dental implants [9]. One of the most important factors that cause the failure of dental implants is marginal bone loss around the implant. Early bone remodeling is a non-infectious remodeling process in the first year after implant placement. Factors affecting it include surgical reasons (improper implant position, excessive heating of the bone during the preparation of the implant site, features of the implant module crest, excessive compression of the cortical bone around the implant) and prosthetics (type of connection) implant/abutment, presence and location of implant/abutment microgap, abutment height, remaining cement, initial loading), creation of biological width, one-stage or two-stage implant placement, micron movement of abutment and placement and remove of abutment [28]. Among other factors affecting marginal bone loss, we can mention the vertical height of the implant placement relative to the bone (crestal or subcrestal), restoration, soft tissue thickness, and gingival height of the abutment [22, 28–30].

Due to the importance of marginal bone loss on implant success and the increasing use of dental implants, researchers have tried to investigate and control factors affecting marginal bone loss.

The vertical position of the implant relative to the crest is one of the factors that affect marginal bone loss, and there is still no consensus [31–34]. According to the investigations carried out in this study, the amount of marginal bone loss in the crestal position three months after implant placement (p-value: 0.048) and three months after implant loading (p-value: 0.001, as a mean) was less than the implants placed in the subcrestal position. One of the causes of marginal bone loss is the presence of microorganisms in the microgap between the implant platform and the abutment. In the subcrestal technique, the marginal bone loss was higher due to the depth of this microgap in the bone. Some studies have shown that bone loss in supracrestal implants is less than crestal, which is in line with the confirmation of this theory [10].

In line with the results of our study, Kim YT and colleagues in 2017, in a review of 143 implants, concluded that implants that were lower than the marginal bone (subcrestal) significantly had more fractures than They were related to crestal implants and higher than crestal implants [31]. Hermann et al. suggested that marginal bone loss could be influenced by the vertical position of the microgaps [35]. It has been reported that microgaps placed above the crestal bone may prevent changes in the marginal bone surface [36].

The result of the study by Pellicer-Chover H and his colleagues, which was conducted on 23 implants, showed that marginal bone loss in subcrestal implantsis more than in crestal implants, like the results of our study [33]. In a histological study, Piattelli and his colleagues evaluated the bone response to different microgap locations relative to the crestal bone (implants that were placed 1–2 mm above the crestal bone, implants that were placed at the surface of the crestal bone, and implants which were placed 1–1.5 mm below the crestal bone) They found that if the microgap was moved away from the crestal bone, minimal bone loss and minimal inflammatory infiltration occurred [37]. In 2023, G Paolantoni and colleagues investigated the influence of crestal and sub-crestal implant position on development of peri-implant diseases: a 5-year retrospective analysis. Patient-based analysis showed a 25.6% of peri-implant mucositis and 32.6% of peri-implantitis for implants placed with the shoulder in crestal position, while for implants inserted in sub-crestal position the percentage of peri-implant-mucositis and peri-implantitis were 19%. The outcomes of this study clinically demonstrated that a deep position of implant shoulder did not provide any benefits. On the contrary, it may be considered a possible risk indicator for implant diseases [38], which is in line with the results of our study.

The studies of de Siqueira RAC et al. and Al Amri MD et al. had different results. In these studies, which examined 78 implants, the amount of bone loss in crestal implants was higher than in subcrestal implants [32, 39]. In a meta-analysis conducted by N Palacios-Garzón et al. in 2019, the results showed that the available data could not be relied upon to reach a definitive conclusion regarding the better vertical crestal or subcrestal position of dental implants and the need to We have more studies [40]. In 2024, Magda Mensi et al. investigated 38 healthy patients were enrolled to receive bone level (BL – Control group) or 2 mm sub-crestal (SC - Test group) conical connection, platform-switched implants. Marginal Bone Modification (MBM) was calculated on standardized radiographs at surgery (T0), loading (T1), and 6 months (T2) and 12 months after loading (T3), the results showed that the Sub-crestal conical connection, platform-switched implants could be a suitable clinical choice to avoid BL with exposure of the treated implants surfaces [41], which is not consistent with the results of our study.

Several prosthetic components can significantly influence on marginal bone loss:

- Implant-Abutment Connection:
  The design of the connection between the implant and the abutment (supporting the crown) plays a crucial role. Studies suggest that external hexagon connections are linked to greater bone loss compared to internal connections [42].
- Abutment Material:
  The material used for the abutment can also influence bone health. Titanium abutments are widely considered the gold standard for minimizing bone loss due to their biocompatibility with the jawbone [43].
- Crown Material:
  The material chosen for the crown can have an impact as well. ZBC (ZrO2-based ceramics) crowns are generally preferred over metal crowns. Zirconium crowns have significant biocompatibility, are non-allergic, and have high compressive strength, high tensile stress, and good aesthetics. Because of its superior mechanical properties, zirconium is mainly used in high stress areas such as the molar region [44, 45].
- Fit of the Prosthesis:
  A well-fitting prosthesis (the entire restoration, including implant, abutment, and crown) is crucial. Micromovement within a poorly fitting prosthesis can irritate the gingiva and contribute to bone loss [45].

Additional factors affecting marginal bone loss:

While prosthetic components play a significant role, other factors also influence marginal bone loss:

- Oral Hygiene: Poor oral hygiene allows plaque and bacteria to accumulate, leading to inflammation around the implant and subsequent bone loss.
- Smoking: Smoking is a major risk factor for peri-implantitis, an inflammatory infection that can lead to bone loss around the implant.
- Medical Conditions: Certain medical conditions, like diabetes, can also increase the risk of bone loss around dental implants [46].
- Restorative Protocol:

Pre-surgical planning: Utilize digital radiographs and 3D scans for accurate implant placement and minimal bone drilling [47].

Minimally invasive surgical technique: minimally invasive surgical techniques to reduce surgical trauma and promote faster healing, minimizing bone resorption. The average survival rate is 97.0% (range, 90–100%) for the flapless procedure and 98.6% (range, 91.67–100%) for the flap procedure but the study showed that the survival rate between the two interventions is not statistically significantly different [48].

Delayed loading protocol: After implant placement, allow for a sufficient healing period (typically 3–6 months) before applying functional load. This allows for optimal osseointegration and minimizes bone resorption, The risk of early bone loss in the immediate loading group was higher than that in the delayed loading protocols group. For removable prostheses and nonsplinted implants, delayed loading protocols was preferred [49].

From a biological view, the subcrestal placement of the implant may cause the inevitable microgap of the implant-abutment junction, which is always surrounded by inflammatory cells, away from the outer edge of the implant and the adjacent bone. It can facilitate the connection between the neck of the implant and the bone [50].

The thickness of the soft tissue around the implant is another important factor affecting the amount of marginal bone loss. This tissue acts as a barrier that protects the bone and structures around the implant against microorganisms and external trauma, and if the thickness of the soft tissue around the implant is less, marginal bone loss will occur until the biological width is created. Studies have shown that if the gum type is thin (less than 2 mm thick), the amount of marginal bone loss will be higher [51–53]. In natural teeth, the average biological width from the sulcus base to the alveolar bone margin was 2.04 mm, of which 0.97 mm is the junctional epithelium and 1.07 mm is connective tissue [54].

Mucous attachment around implants or during wound healing after implant placement and abutment placement are established [55]. The mechanism involves epithelial proliferation followed by collagen fiber organization and may take several weeks to complete. This adhesion is called “biological width” and acts as a seal to protect the hard tissues around the implant [56]. Recently, the biological width around dental implants placed at the crestal level has been measured in human histological studies. The vertical dimensions vary from 3.26 to 3.6 mm, representing the minimum space required to create optimal flooding and protect the underlying tissue from external factors. When the vertical space is insufficient to create biological width, the healing process includes marginal bone loss [57, 58]. Linkevicius and his colleagues, Linkevicius, Puisys, Steigmann, and also Suárez-López Del Amo, concluded that implants that are initially placed with thicker soft tissues around the implant show better bone stability [52, 59, 60]. In Aliye Akcalı’s 2017 systematic review, they concluded that there was insufficient evidence to answer the question of the difference in clinical outcome in terms of marginal bone loss between implants placed in sites with an initial soft tissue thickness of less than 2 mm and those with 2 mm and more are placed. In addition, well-designed controlled clinical studies are needed to analyze the effect of soft tissue thickness on the clinical outcomes of dental implants [61].

According to Eriberto Bressan’s 2023 Revo systematics, the soft tissue around the implant creates a biological barrier against bacteria and protects against peri-implantitis. Soft tissue thickness appears to influence marginal bone loss after a short follow-up period. Based on this systematic review, more studies are needed to evaluate the effect of soft tissue thickness on marginal bone loss [30].

The results of our study also showed that the amount of marginal bone loss in soft tissue thickness of 2 mm and less than 2 mm in three months after implant placement (P-value: 0.01) and three months after implant loading (P-value: 0.001), it is significantly more than the soft tissue thickness more than 2 mm. Also, the highest marginal bone loss was in the subcrestal group with soft tissue thickness less than 2 mm in three months after implant loading (-1.58 mm average), and the lowest marginal bone loss was in the crustal group with soft tissue thickness greater than 2 mm and in three the month after implant placement was (-0.11 mm average).

The limitations of this study can be mentioned below:

- Inability to measure buccal and lingual bone loss in two-dimensional radiography.
- Short-term follow-up period.
- Not using different implant systems.
- Not placing the implant in the form of a split mouth.

## Conclusion

The relationship between implant’s vertical position, soft tissue thickness and marginal bone loss is complex. While this research suggests that subcrestal placement and thinner tissues potentially lead to greater bone loss, more research is needed to fully understand the individual and combined effects of these factors.

The time factor also significantly affects the amount of marginal bone loss, so the loss in the third month after implant placement is more than the loss in the third month after implant placement (six months after implant placement).

## Data Availability

The datasets used and analyzed in this study are available on reasonable request from the corresponding author (shahsavand.leila@yahoo.com).

## Abbreviations

CBCT: Cone beam computed tomography
SPSS: Statistical package for social science
GBR: Guided bone regeneration
